# Options for design of real-world impact studies of single-dose vaccine schedules

**DOI:** 10.1016/j.vaccine.2018.02.002

**Published:** 2018-08-06

**Authors:** Silvia Franceschi, Gary M. Clifford, Iacopo Baussano

**Affiliations:** a Cancer Epidemiology Unit, Aviano National Cancer Institute IRCCS, Via Franco Gallini 2, 33081 Aviano (PN), Italy; b International Agency for Research on Cancer, 150 cours Albert Thomas, 69372 Lyon Cedex 08, France

**Keywords:** HBV, hepatitis B virus, HICs, high-income countries, HPV, human papillomavirus, LMICs, low- and middle-income countries, OE, overall effectiveness, STI, sexually transmitted infections, VLP, virus-like particle, Cervical cancer, Human papillomavirus, Low- and middle- income countries, Single-dose, Vaccination, Effectiveness

## Abstract

Based on existing evidence for efficacy, savings, and advantages in delivery, some countries may elect to pilot or roll out single-dose human papillomavirus (HPV) vaccination (instead of, or in combination with, two-dose) in advance of a WHO policy decision. Accelerated evidence of population-based effectiveness (hereafter referred to as overall effectiveness, OE) of one-dose vaccine programs could be gained through regular surveys of HPV prevalence in young women before and after vaccination introduction. In order to offer the earliest information on OE, one-dose HPV vaccination should target one or more birth cohorts as close as possible to the age when sexual activity most often starts in a given population. A catch-up one-dose vaccination program of girls up to 18 years of age who would have been too old to profit from the introduction of a routine HPV vaccination program in preadolescents would minimize the interval between vaccination and the possibility to monitor vaccination impact in young women. In addition, catch-up is especially desirable in low- and middle-income countries with little access to screening as “missed” cohorts may face high risk of cervical cancer death. HPV prevalence should be firstly monitored in age groups of women who may already be sexually active but still reluctant to admit it and to accept vaginal examination for the collection of cervical cells. Hence, HPV testing from urine samples, for which good concordance with cervical cells has been proven, offers a feasible approach to assess periodically vaccine OE in representative samples of 17–20 year-old women. This type of observational study would greatly benefit from the presence of a population census and the creation of a vaccination registry. A real-world demonstration of OE of the new schedule would complement the findings of ongoing clinical trials and immunogenicity studies on the efficacy of single-dose HPV vaccination.

## 1 Introduction

Since proof of efficacy of virus-like particle (VLP) vaccines against human papillomavirus (HPV) became available, there was strong determination to make the time-window between the commercial release of HPV vaccine and access for populations at high-risk of cervical cancer in low- and middle-income countries (LMICs) shorter than it had been for the recombinant hepatitis B virus (HBV) vaccine, the only other cancer-preventive vaccine, which had taken nearly 30 years to be introduced in the majority of LMICs [1].

The HPV vaccine was first marketed in 2006 and by the early 2010s national HPV vaccination programs had become available in many high-income countries (HICs). Two landmark events led to widening of vaccine access in LMICs: (1) the GAVI Alliance started to support HPV vaccine introduction in 2013, and (2) in 2014, the non-inferiority of two doses compared with three led to a two-dose schedule being endorsed by WHO in 2014 in girls below age 15 [2]. Several dozens of demonstration projects of HPV vaccination were launched in LMICs, which have been recently summarized [3], [4]. Nevertheless, few LMICs, notably Panama, Bhutan, Rwanda, Botswana, South Africa and Malaysia, have so far succeeded in the implementation of national programs. Hence, after more than 10 years since HPV vaccine licensure, access to HPV vaccination remains limited especially in Africa and Asia [5] and the original GAVI target to vaccinate 30 million girls by 2020 is considered at risk [6] (http://www.gavi.org/).

Barriers to national vaccine uptake in LMICs (see LaMontagne et al. in this volume), and how single-dose HPV vaccination could be transformational (Table 1) are widely discussed in other articles in this supplement. However, it is clear that in the coming years, the possibility of a one-dose schedule would greatly facilitate national upscale in those countries that have previously performed demonstration projects, as well as encouraging HPV vaccine introduction in other LMIC that have found it challenging to date. While the most important advantage would obviously be diminishing the cost of purchase and delivery of the vaccine, new types of HPV vaccination programs and immunization campaigns may also become more cost-effective (Table 1).

**Table 1:** Expansions of HPV vaccination programs facilitated by single-dose schedule.

Possible expansions	Obstacles	Contribution of single-dose
National up-scale	High cost of vaccines after GAVI support ends	Half price
Broader age target	High cost, difficult to vaccinate out-reach girls >1	Better feasibility and savings
Multiple delivery systems	School-based delivery is the best but not possible in all cases	Facilitates delivery in health facilities and mass campaigns, possibly combined with other vaccines
Gender-neutral program	High cost, less cost-effective than girls	Savings, better herd immunity and program robustness
Vaccination in childhood	Very busy vaccine schedule	Savings and logistically easier

The strongest evidence for the efficacy of one-dose vaccination will come from ESCUDDO [7], a population-based randomized trial in which one and two doses of either 2-valent or 9-valent HPV vaccines will be compared (see accompanying article by Kreimer et al.). However, the outcome of that trial is not expected before 2023. In the meantime, some countries may elect, based on existing evidence for efficacy [8] and operational advantages, to pilot one-dose vaccination, in advance of a WHO policy decision. We here describe research study designs which could provide, in a few years and in parallel with ongoing clinical trials, the first real-life evidence of the effectiveness and operational advantages of one-dose HPV vaccination.

## 2 Study design options

Monitoring HPV vaccine impact is complicated by the buffer period, i.e., the time interval between HPV vaccination of preadolescent girls and evidence of impact on cervical cancer and pre-cancer, and by the lack of a serological test providing a threshold for protection. Hence, in the near-term (5–10 years), the only informative outcome is to measure post-vaccination changes in type-specific HPV infection prevalence in populations of young women, as recommended by a WHO expert group [1].

With respect to accelerated impact data from LMICs, we excluded certain study design options. Firstly, we assume that it would be considered unethical to randomize women, individual- or cluster-wise, to no HPV vaccination. Secondly, we assume it unlikely that any randomized non-inferiority trial comparing one versus multi-doses of vaccine could be (or should be) put into place in parallel to the ESCUDDO trial. Finally, immunogenicity bridging studies are useful but not conclusive as it is already known that one-dose produces lower HPV antibody levels than multiple-doses but the clinical implications of this inferiority are unclear [9].

Rather than discuss the aforementioned study designs we will focus on how to best use observational studies in which type-specific HPV prevalence in young women is monitored through repeat HPV surveys before and after the introduction of one-dose vaccination. HPV prevalence in unvaccinated and vaccinated cohorts will then be compared to assess the real-world overall effectiveness (OE, i.e., the population-level impact of one-dose vaccination programs) [10], [11].

### 2.1 Repeat surveys of HPV prevalence

The earliest evidence of the population-level impact of three-dose HPV vaccination from high-income countries (HICs) has been obtained by comparing type-specific HPV DNA prevalence in young women before and after vaccination [12]. We are using a similar monitoring approach which has produced similarly encouraging results in Bhutan and Rwanda, the first LMICs to introduce national HPV vaccination in 2010 and 2011, respectively [13]. Whilst monitoring protocols would need to be adapted to given settings, key elements of an adequate evaluation of the effectiveness and operational advantages (i.e., decreases in vaccine and delivery costs and possible expansion of reachable sub-populations in terms of age range and possibly gender) of one-dose vaccination are summarized in Table 2 and discussed in more detail below.

**Table 2:** Key elements of an evaluation of single-dose catch up vaccination.

Requirement	Aim
No previous multi-dose HPV vaccination	To avoid confounding from multidose vaccination in other girl cohorts (herd immunity)
Sufficiently large and stable population	Vaccination of ≥40,000 adolescent girls
Population census	To monitor coverage and select random samples
Vaccination registry	To distinguish vaccination status more accurately and enable follow-up studies
Health system records	To estimate costs and logistics of vaccine delivery
Broad single-dose catch-up vaccination	Vaccination up to ages as close as possible to sexual debut e.g. 12–17 years while still unlikely to have been already infected by HPV^a^
High coverage	To optimize the comparison of birth cohorts of unvaccinated and vaccinated girls
Serial HPV urine surveys, pre and post vaccination	To monitor earliest vaccine impact in HPV prevalence in population-based representative samples of youngest women (e.g., 17–19 year female students)
Repeat HPV cell surveys, pre and post vaccination	To monitor medium term vaccine impact on HPV prevalence in representative population-based samples of fully sexually active age groups of women (e.g. ≥ 25 years) and/or in sentinel high-risk groups (<25 years)

a Single-dose vaccination of older girls may coexist with “routine” vaccination with 1 or 2 doses in preadolescents.

### 2.2 Location and timing of one-dose vaccination studies

The choice of the location for a demonstration study of one-dose vaccination against HPV in LMICs firstly depends on the willingness of local political and health stakeholders to endorse the initiative and share with the population the motivations that have led to it. In addition, it is essential that the birth cohorts of girls who will receive the one-dose schedule are not going to be protected by the indirect effectiveness of previous two- or three-dose vaccination programs as this may substantially inflate the measured OE (Table 2). Along the same lines, it is important that the study population be stable, i.e., the HPV transmission dynamic is unlikely to be affected by substantial arrival of vaccinated or unvaccinated individuals from other places. Indeed, a geographically isolated population would be ideal but also a population dwelling in a region in a rather large LMIC which had already carried out a local demonstration project of multiple-dose HPV vaccination would be acceptable provided the two areas were sufficiently far apart. Other desirable characteristics of the study location would be the availability or feasibility of establishing a HPV vaccination registry to track vaccination coverage overall and by birth cohort and village, and allow long-term follow-up of vaccinated girls and linkage studies with the screening registries. A sufficiently well-organized health system would also be an asset to allow the collection of delivery costs, wastages, several adverse reactions, etc.

Irrespective of the chosen location(s), the timing of the study is essential to be able to produce results earlier or in parallel with the release of the first efficacy data on one-dose schedule that is expected in 2023 (see Kreimer et al. in this volume). It would therefore be essential to carry out the baseline HPV survey of unvaccinated 17–20 year-old women no later than 2018. The timely performance of a baseline survey has been challenging in countries who implemented multiple-cohort programs, i.e. routine vaccination of preadolescents and of catch-up of older girls. Very successful vaccination programs in Bhutan [14] and Rwanda [15] were, for instance, put in place so quickly that when we were able to do the first urine survey in 2013, 90% and 40%, respectively, of the participants had already been vaccinated [13]. We were therefore able to compare vaccinated girls to unvaccinated girls being aware that the decrease in HPV prevalence among vaccinated girls specifically reflected the impact of catch-up vaccination as in both countries the age at vaccination among vaccinated survey participants was on average 16 years [13].

In the next two sections, we will describe in more detail the type of study that we consider the most propitious for producing timely and robust real-world data on the effectiveness and operational advantages of one-dose vaccination, i.e., catch-up vaccination studies.

### 2.3 One-dose catch-up vaccination

The buffer period, the time interval between the introduction HPV vaccination and the earliest evidence of OE depends on the age group targeted by the vaccination and the average age at which the risk of being infected by HPV, rises very steeply, i.e. after the beginning of sexual intercourse. Of note, HPV infection can also be acquired through non-penetrative sexual intercourse. While HPV vaccination is most efficacious in pre-adolescence, the younger the age at vaccination (and the older the average age at sexual debut in a country), the longer will be the waiting for evidence of vaccine impact.

Conversely, if one-dose vaccination is provided to older girls, the time needed to assess OE would be substantially shortened by the proximity of possible sexual exposure to HPV. However, the target age-range for vaccination is population-specific as the vaccination of a sizeable fraction of sexually active and potentially already infected girls would lower the estimated OE particularly in short-term studies. In addition, catch-up is highly desirable in LMICs with little access to screening as “missed” cohorts may face high risk of death from cervical cancer. Mathematical models [16], [17] and survey data [12], [13] have demonstrated that up to age 18 or so, catch-up can be highly cost-effective in anticipating the benefits of the intervention and, as vaccine price diminishes, catch-up vaccination is increasingly encouraged, e.g., by WHO [18] and GAVI.

**Fig. 1:**
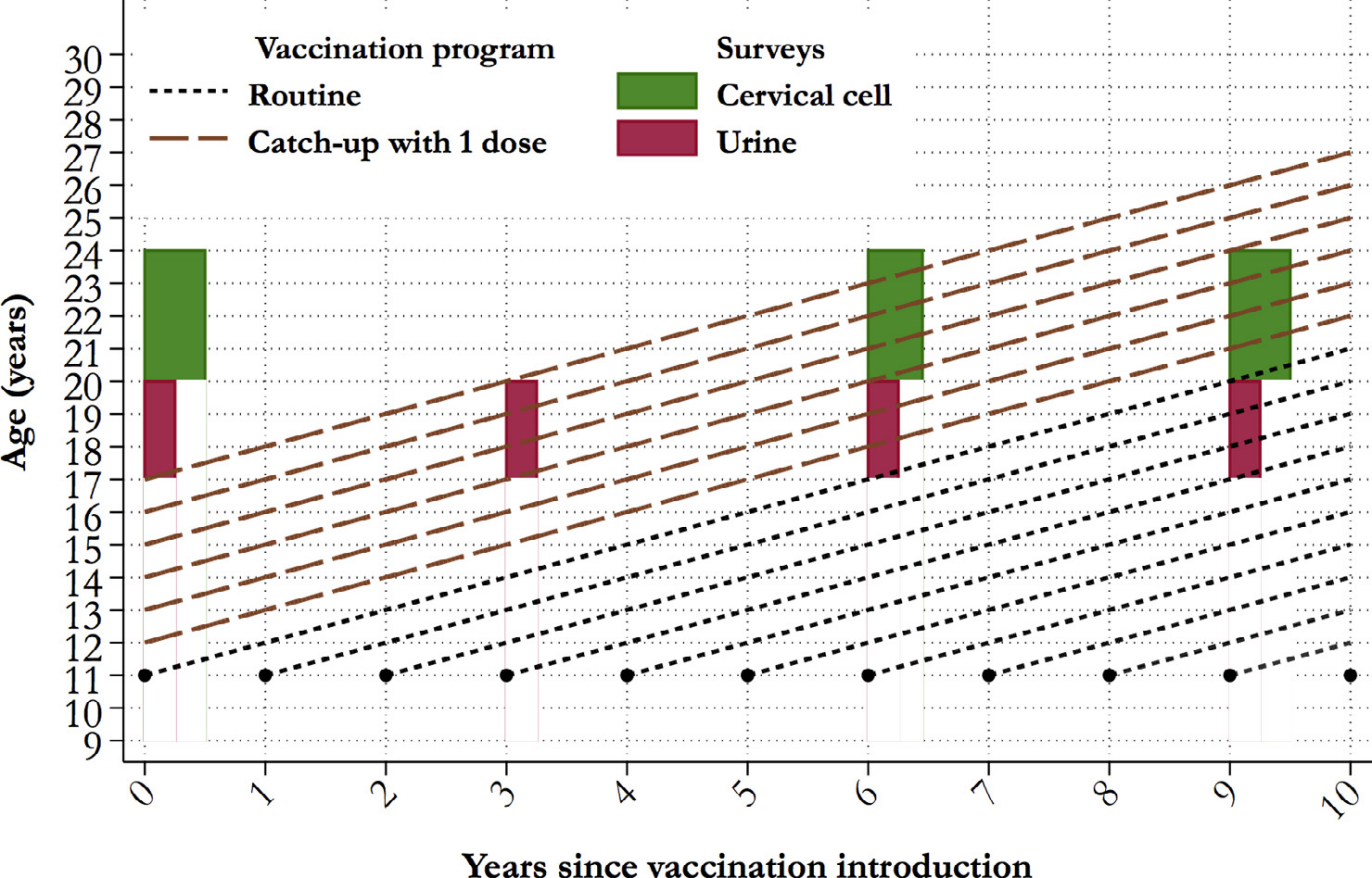
Lexis diagram of a one-dose HPV vaccination study including catch-up with one dose and routine vaccination with one or two doses and repeat HPV surveys.

Fig. 1 shows the possible time framework of a study of HPV prevalence prior to and after the beginning of one-dose vaccination of multiple cohorts of girls aged, in our example, 12–17 years. We envisage that one-dose catch-up vaccination will target girls aged 12–17 years in 2018, i.e., cohorts born in 2001–2006, and should be accompanied by a “routine” vaccination of 11 year-old girls (or equivalent school grades) using a two-dose or, if deemed acceptable and convenient by political and health stakeholders, a one-dose schedule. This combined strategy should be attractive for one or more LMICs that have become convinced of the importance of HPV vaccination and may be willing, if technically supported, to offer vaccination to a larger number of girls at the same cost as a smaller program.

For monitoring purposes we propose to carry out two different types of HPV surveys: (1) urine-based surveys (in red in Fig. 1) that better suit unmarried and younger women, i.e., 17–20 years of age; and (2) surveys based of cervico-vaginal samples (in green) that are more acceptable by older sexually active women (see the section on Choice of biological samples). The actual age-range of routine and catch-up vaccination and the timing of the different surveys will need of course to be finely tuned to the local context in which the study will be conducted, but baseline surveys will have to be carried out prior to, or at the very beginning of, the catch-up program. Provided that catch-up can rapidly achieve a high coverage (≥50%), repeat urine surveys to capture the earliest decreases in HPV prevalence in 17–20 year-old women who have had access to the one-dose schedule is already informative at year 3 after the introduction of vaccination. Follow-up surveys of older women using cervico-vaginal cells could start including a substantial proportion of vaccinated women at year 6 and thereafter be aligned with the urine surveys at regular intervals for as many years as possible. If HPV surveys were sustainable for 9 years or more, the monitoring program would eventually also produce the first estimates of the OE of the combined impact of routine and catch-up vaccination in the study population (Fig. 1).

### 2.4 Statistical methods

The range and size of birth cohorts which needs to be targeted by one-dose vaccination needs to be large enough to affect the circulation of HPV infection in the study region/country. Pragmatically, and depending on the stability of the population in the selected region, at least several tens of thousands of 12–17 year-old girls should receive the one-dose schedule. For example, approximately 40,000 girls were targeted by the initial national catch-up program in Bhutan [14] and by the largest GAVI-supported HPV demonstration projects [3].

The number and age range of young women who need to be monitored to have a sufficient power to measure OE is significantly smaller than the population targeted with one-dose vaccination. The minimum size of each survey depends on the prevalence of HPV in young women (which can be unknown when the study begins) and the age range at which sexual activity in the targeted group starts (which can be usually known from national statistics). Additional sources of uncertainty are the efficacy of single-dose vaccination and the coverage attainable in the study area [19]. Conversely, the herd effect, i.e., the protection that a sufficiently widespread vaccination provides to unvaccinated individuals, should not be of immediate concern. Regardless of the vaccination schedule, it should not operate immediately among the recipients of the catch-up program [20] as they will begin sexual intercourse years earlier than the girls included in the routine vaccination (Fig. 1).

**Fig. 2:**
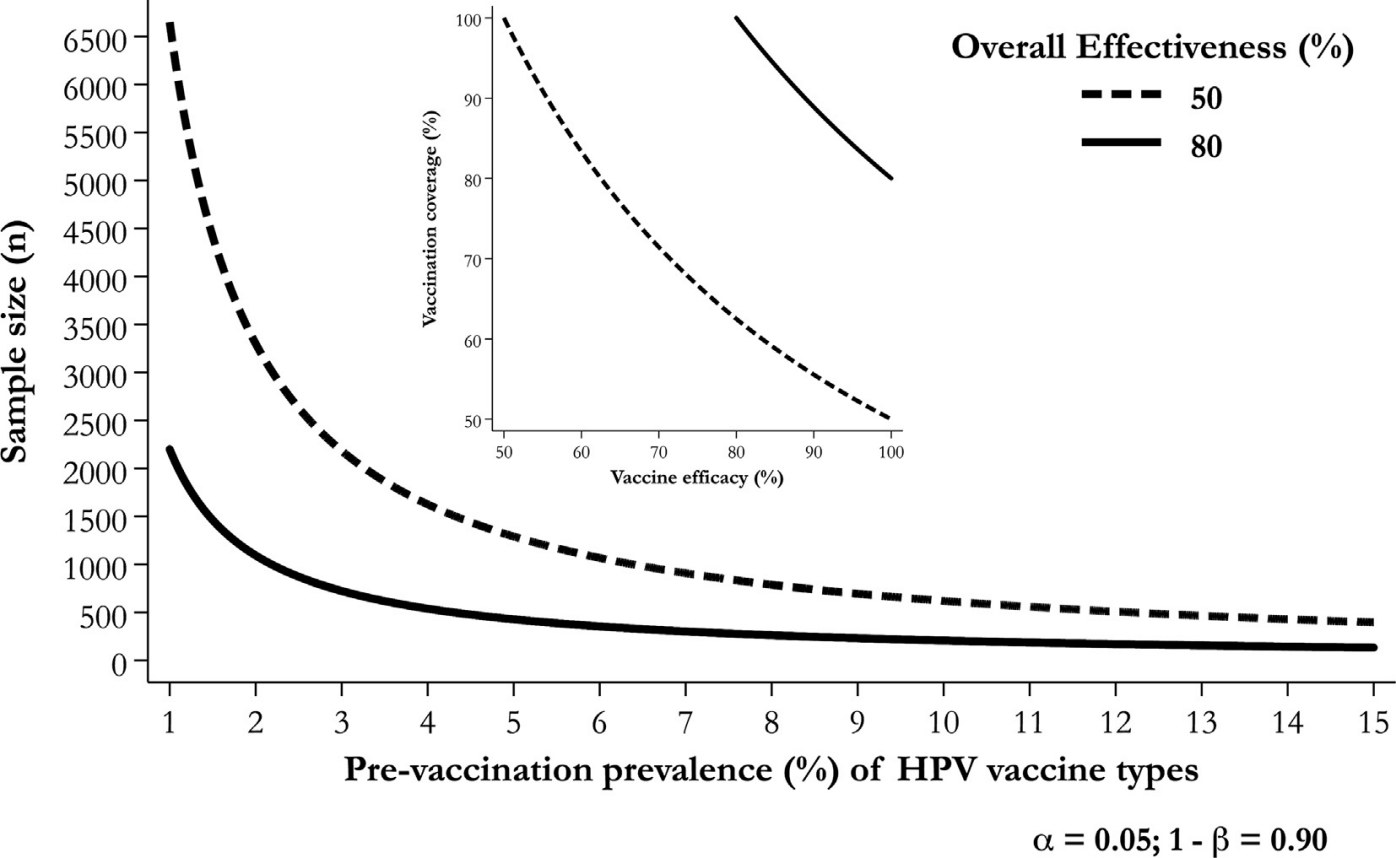
Effectiveness of HPV vaccination detectable according to sample size and pre-vaccination prevalence of vaccine HPV types women aged 17–20 years. The inset figure shows overall effectiveness as a function of vaccine efficacy and coverage.

Fig. 2 shows that the minimal sample size required, with α = 0.05 and β = 0.90, to detect OE (i.e., 1 minus the ratio of vaccine HPV type prevalence in a repeat survey to the corresponding prevalence in the baseline survey) of 50% or 80%. These percentages are chosen as mere examples of two plausible scenarios. As shown in the inset figure, under the assumption of temporary absence of herd protection, OE simply depends on the product of efficacy of a one-dose schedule and the coverage among catch-up-targeted cohorts (both assumed here to be ≥50%). Either very good coverage (e.g., 90%) and moderate efficacy (e.g., 54%) or moderate coverage and very good efficacy may then provide 50% OE. The more complete the coverage the closer are efficacy and OE. For example, if HPV 16/18 prevalence in the urine of unvaccinated 17–20 year-old women were each 2.5%, as we observed in Rwanda [13], at least 2500 participants *per* survey would be necessary to detect a 50% overall OE. The number of women required would increase steeply if pre-vaccination prevalence of vaccine types were lower than 2% but it would be smaller if OE were 80%, e.g., if both one-dose efficacy and the coverage were ≥89%.

The OE of the one-dose schedule in our present example will be mainly estimated by comparing the prevalence of vaccine-targeted HPV types in unvaccinated and vaccinated birth cohorts; that is, by comparing the prevalence in baseline and repeat HPV surveys. The comparison of vaccinated and unvaccinated birth cohorts somewhat corresponds to “by intention to vaccinate” analyses in randomized clinical trials. If coverage is sub-optimal, vaccinated and unvaccinated women in a repeat survey can also be compared [13]. An accurate history of HPV vaccination is therefore very important. The information can be obtained from the survey questionnaire but it would be greatly improved by cross-checks with vaccination registries if available.

Importantly, the validity of estimates of OE will be reliable only if participants in all surveys are recruited in a very consistent way, i.e., if they are comparable with respect to age, place of recruitment (schools, type of health clinic, home, etc.), place of origin/living, socio-economic level, and most challenging, sexual habits (see also section on “Additional and ancillary studies”). The need to collect short but accurate questionnaire-based information on all the characteristics that are or may be associated with the probability of an early acquisition of HPV infection cannot be exaggerated. This information will show if indeed similar young women have been recruited and allow us to also perform between-survey comparison after stratification and adjustment for relevant characteristics. Of note, the need to perform a priori defined sub-group analyses of HPV prevalence will necessitate an *ad hoc* increase in survey size compared with the minimum requirements.

While very detailed sexual questions are neither feasible nor acceptable in surveys of young women in LMICs, bias in the report of sexual activity can be attenuated by skilled female interviewers or by self-filled questionnaires in combination with strict assurance of questionnaire anonymity [13]. In addition, possible differences in sexual behavior and hence HPV exposure over time could also be objectively explored by assessing the prevalence of HPV types other than those against which the vaccine is efficacious and in the prevalence of other sexually transmitted infections (STI). For example, PCR-based testing for *Chlamydia trachomatis* can easily be added to HPV testing in the same sample, as we did in Bhutan and Rwanda [13].

### 2.5 Choice of biological samples

The gold-standard samples for the detection of current HPV infection are clinician-collected cervico-vaginal cell samples. Indeed, most of the existing assessments of HPV vaccine impact in HIC have been based on repeat surveys using this approach [12], and greatly aided by existing gynecological sampling infrastructure for cervical cancer screening. This design has also been used to characterize HPV infection in unvaccinated populations prior to HPV vaccine roll out in Bhutan [21] and Rwanda [15] and many other IARC surveys [22]. This kind of approach is well accepted and can be representative of the general female population, in age groups of well-established sexual activity, e.g., in women older than 20 or 25 years or, in many LMICs, where premarital intercourse is stigmatized, in married women only.

However, in order to obtain the earliest evidence of OE of one-dose HPV vaccination, HPV prevalence should be measured in younger often unmarried women who may or may not have started or be willing to admit sexual intercourse. Women in this age group are often reluctant to accept a gynecological examination for the collection of cervical cells, also because gynecological exams and cervical screening have little or no clinical value to them. Of note, HPV-based screening is not recommended below age 30, due to the high prevalence of HPV infections that will rapidly clear spontaneously [23].

The settings where gynecological exams are offered to women below 25 years of age are mainly STI clinics and reproductive health clinics that attract very sexually-active women often at increased risk of HPV infection. These settings offer therefore a special opportunity for “sentinel” surveys with extra statistical power, due to high HPV prevalence, but at the cost of a potentially poor representativeness of the general female population of the same age [24], [25], [26]. Utmost care is needed, therefore, to make sure that the combination of young women included in repeat surveys is well comparable with respect to sexual behavior.

Self-collected cervico-vaginal samples have proven to be nearly equivalent to clinician-collected samples for HPV-based cervical screening and are also a valid option for monitoring HPV vaccine impact. The predominant advantage of self-collected cervico-vaginal samples especially in LMICs is the high throughput collection due to much lower requirements for specialized personnel and disposable plastic equipment (specula, etc.). In addition, it has been shown in many settings that the acceptability is better for self-collected than for clinician-collected samples [27], [28] and possible in some settings also among unmarried young women [29].

A very powerful alternative to cervico-vaginal samples to evaluate HPV prevalence is represented by urine sample. Not only is collection of urine samples a well-accepted non-invasive procedure but many previous problems in the storage and processing of this type of samples have been overcome [13], [30], [31]. Systematic reviews have shown a good concordance with cervico-vaginal cells for HPV positivity in women [32]. Indeed, when performant DNA extraction protocols are combined with highly-sensitive PCR-based assays, HPV prevalence can be even higher in urine than in cervico-vaginal samples obtained from the same women [33], [34]. Although laboratory protocols for HPV detection in urine samples are somewhat more demanding, in terms of equipment and sample handling than those for cervical cells, urine has enormous advantages in terms of feasibility and representativeness of sample collection in young women.

A good demonstration of the feasibility of urine collection in LMICs is provided by the two similar urine surveys that we performed in 2013 in high-school students in Bhutan and Rwanda. Girls aged 17–20 used a urine self-collection device designed to immediately mix first void urine with a preservation medium [13]. Self-collection of urine proved to be highly acceptable in both settings, and also allowed a high-throughput of sample collection with minimal requirements for trained staff. Collections kits were distributed in schools to hundreds of girls at a time and the vast majority of girls collected first-void urine at their home and returned samples at the beginning of the following school day with a very good subsequent yield of DNA [13]. Indeed, urine sampling in order to establish HPV prevalence has even been proven acceptable in school girls down to the age of 11 years [31]. Of course, when sampling girls who are not all sexually active, HPV prevalence will be lower than at older ages, requiring larger sample sizes to observe similar efficacy. In populations where social norms delay the sexual debut of young women for a few additional years, the upper age limit of urine-based surveys could be raised up to age 25 years, by which age most women should be sexually active [35].

### 2.6 Additional and ancillary studies

It might be possible to apply study designs that use one-dose vaccination both during catch-up and in routine activity, the decision to give or not a second dose being based on the results of future efficacy studies on one dose. Such an approach may be referred to as an extended interval two-dose schedule (e.g., 0 and 60 months), similar to the approach formerly adopted to evaluate and accelerate the transition from a three- to two-dose schedule [2]. However, a safety window of 5 years before deciding whether to withhold a second dose would probably not apply to girls vaccinated up to 17 years-old and, by and large, recalling girls for the administration of a second vaccine dose would be challenging in LMICs.

Aside from the urgency of understanding the early OE and operational advantage of the one-dose schedule using highly feasible and relatively inexpensive school-based urine surveys and clinic-based cervical cell surveys, increasingly complicated tools can be conceived to monitor changes in HPV prevalence before and after vaccination more accurately. In the presence of a fairly good enumeration of the study population by sex, age, and place of living, for instance, random samples of young women could be used to measure HPV prevalence.

A large demonstration project of one-dose vaccination may also be the opportunity to create a platform for systematic monitoring of HPV vaccination in one LMIC or more. A computerized HPV vaccination registry may be established and eventually allow the evaluation of one-dose OE and the partitioning of OE into direct effectiveness and indirect (herd) protection based on cohort- or nested case-control analyses. It may be subsequently used also to assess additional vaccination strategies or the combination of vaccination with HPV-based screening programs. Finally, some parallel investments may be worth considering including the creation of biobanks of urine, cervical cells or blood samples linkable to vaccination registries and medical data.

## 3 Conclusions

The project outlined here rests on the opinion that one-dose HPV vaccination may well be a landmark in the achievement of global access to a cancer-preventing vaccine and in a better understanding of the way VLP-vaccines and vaccine adjuvants work. Of course, other obstacles to universal HPV vaccination, such as the reluctance of stakeholders to invest in primary prevention whose return will only be appreciable after decades, unjustified fears of vaccines, and the lack of manufacturers in LMICs, will remain but they will be eased by the decrease in vaccine cost and logistic challenges.

Building upon our experience in monitoring the burden of HPV infections and the effectiveness of HPV vaccination in both HICs and LMICs, our present article endorses the feasibility and value of carrying out a sufficiently large one-dose vaccination program of girls up to 18 years of age and repeat HPV surveys in young women. This would provide the earliest real-world evidence of the effectiveness and operational advantages of one-dose HPV vaccination, which, if consistent with that from trials and studies described in other chapters of this volume, would justify and accelerate a major change in global HPV vaccination policy.

## Conflicts of interest

None.
